# Clamshell and Hemiclamshell Incisions for Complex Intrathoracic Tumors: A Retrospective Analysis of Indications and Outcomes

**DOI:** 10.1111/1759-7714.70373

**Published:** 2026-08-18

**Authors:** Marco Mammana, Vincenzo Verzeletti, Giulia Pagliarini, Alessandro Bonis, Viola Sambataro, Federico Rea, Andrea Dell'Amore

**Affiliations:** ^1^ Thoracic Surgery Unit and Lung Transplant Centre, Division of Surgery University–Hospital of Padua Padua Italy; ^2^ Department of Cardiac, Thoracic, Vascular Sciences and Public Health University of Padua Padua Italy

**Keywords:** clamshell, hemiclamshell, intrathoracic tumors, thoracic incisions

## Abstract

**Introduction:**

Clamshell and hemiclamshell incisions are uncommon approaches in thoracic oncology. This study aims to describe indications and outcomes of surgical resection using these two incisions and to propose criteria that may prompt their use.

**Methods:**

We retrospectively reviewed patients undergoing tumor resection via clamshell or hemiclamshell incision between 2009 and 2024. Patient characteristics and outcomes were descriptively analyzed.

**Results:**

Clamshell and hemiclamshell approaches were used in 36 and 55 cases, respectively. Median tumor diameter was 10 cm (IQR: 6.4–14.0). Pneumonectomy, vascular resections, and tracheo/bronchoplastic procedures were performed in 41.4%, 33%, and 12.1% of cases; extracorporeal support was required in 11%. Distinct patterns of tumor histology and intrathoracic location were observed between the two approaches. Pneumonectomy and tracheo‐carinal resections were mainly performed through clamshell, while pleural resections were predominantly carried out through hemiclamshell (*p* < 0.05). Postoperative hospital stay was longer in the clamshell group (13 vs. 11 days), whereas other early outcomes were comparable. Overall survival and cause‐specific mortality were similar.

**Conclusion:**

This study provides a descriptive analysis of the clinical use of clamshell and hemiclamshell incisions in thoracic oncology. Distinct indications emerged for each approach, reflecting tumor location and extent. Both techniques were associated with acceptable perioperative outcomes, while long‐term results appeared to be mainly influenced by tumor characteristics. The proposed illustrative algorithm may aid in selecting surgical access for complex intrathoracic tumors.

## Graphical abstract

This graphical abstract summarizes the indications and outcomes of clamshell and hemiclamshell incisions for the resection of intrathoracic tumors. The study provides an overview of tumor histology and anatomical location according to the incision type used. Despite differences in surgical approach, overall survival, and the incidence of cause‐specific death were similar between patients undergoing clamshell and hemiclamshell incisions. These findings suggest that both approaches can be effective surgical strategies for the management of selected intrathoracic tumors, with the choice of incision primarily guided by tumor characteristics and anatomical location.

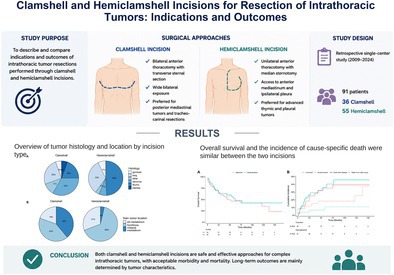

## 1 Introduction

Bilateral anterior thoracotomy with transverse sternal section, also known as clamshell incision, and unilateral anterior thoracotomy combined with median sternotomy, known as hemiclamshell incision, are not standard approaches for intrathoracic tumor resection [1, 2]. The need to resort to such extended incisions may arise in case of bulky masses, in cases where exposure of different regions of the chest at the same time is required, or in cases of proximity or encasement of deeply located vital structures from a tumor.

Owing to their nonstandard nature, cases of intrathoracic tumor resection through either clamshell or hemiclamshell incisions are often the object of single case‐reports [3, 4, 5, 6]. There's only a limited number of surgical case series describing baseline characteristics, surgical indications, and postoperative outcomes of patients operated on through these two approaches. Some of the available studies date back before the advent of minimally‐invasive thoracic surgery [7], others are focused on a single incision and/or a specific histology [8, 9], while others concern specifically the pediatric population [10, 11]. Therefore, it is often difficult for surgeons to understand when it is advisable to use a clamshell or hemiclamshell approach in clinical practice, what are the advantages and disadvantages provided by each of these two incisions and what may be the impact of such extensive procedures on the early postoperative course.

The aims of this study were as follows. First, to provide a comprehensive description of the clinical characteristics and outcomes of a recent, consecutive cohort of patients undergoing intrathoracic tumor resection through clamshell or hemiclamshell incisions. Second, to describe patterns of surgical indications and perioperative variables associated with each approach. Third, to report early and long‐term outcomes in this patient population.

## 2 Subjects

### 2.1 Ethics Statement

This study received the approval by the local Institutional Review Board (approval number 467n/AO/24). The need for individual patient consent was waived due to the anonymized and aggregated form of the data.

### 2.2 Patients and Variables

We retrospectively reviewed all the patients that consequently underwent surgery through a clamshell or hemiclamshell approach for intrathoracic tumor resection at a tertiary thoracic surgery center between January 2009 and June 2024.

Clamshell incision was defined as two bilateral anterior thoracotomies, usually in the 4th intercostal space, joined by a transverse sternal section. Hemiclamshell incision was defined as a unilateral anterior thoracotomy (usually in the 4th intercostal space) connected with a longitudinal median sternotomy. Both surgical incisions are well standardized in the Literature, and a thorough description of the surgical steps can be found elsewhere [1, 2]. When performing hemiclamshell, we did not routinely add a neck incision along the anterior border of the sternocleidomastoid muscle or along the clavicle (sometimes referred to as “trap‐door thoracotomy”), as elsewhere reported [10, 12]. We excluded cases of patients who were approached by a median sternotomy and a separate posterolateral thoracotomy, and cases of patients who underwent surgery through clamshell or hemiclamshell for non‐oncologic reasons (e.g., lung transplantation).

## 3 Methods

We queried the institutional electronic database of operative reports through a word match feature, retrieving all reports in which the description contained the terms “clamshell,” “hemiclamshell,” or respective synonyms.

For each patient we collected baseline variables, characteristics of the disease, details of surgery, and early and late outcomes, including overall survival (OS) and the cause of death.

The tumor size and its prevalent location within the chest were evaluated by reviewing the preoperative computed tomography scan images and by adopting common definitions of the anatomical regions of the chest [13]. The disease stage for both thymic tumors and for non‐small cell lung cancer (NSCLC) was attributed according to the 8th edition of their specific TNM stage classification systems. The severity of postoperative complications was assessed using the Clavien‐Dindo scale: major complications were those with a grade of III (requiring surgical, endoscopic or radiological intervention) or higher [14].

Descriptive statistics were reported as median (interquartile range) for continuous variables and absolute numbers (percentages) for categorical variables. Differences in baseline characteristics and perioperative variables according to surgical approach were explored using the Wilcoxon rank sum test, Chi‐squared test, or Fisher's exact test, as appropriate. OS and disease‐free survival (DFS) were estimated using the Kaplan–Meier method. The cumulative incidence of death from disease and death from other causes was estimated using competing risk analysis; differences according to surgical approach were explored using Gray's test.

## 4 Results

**FIGURE 1:**
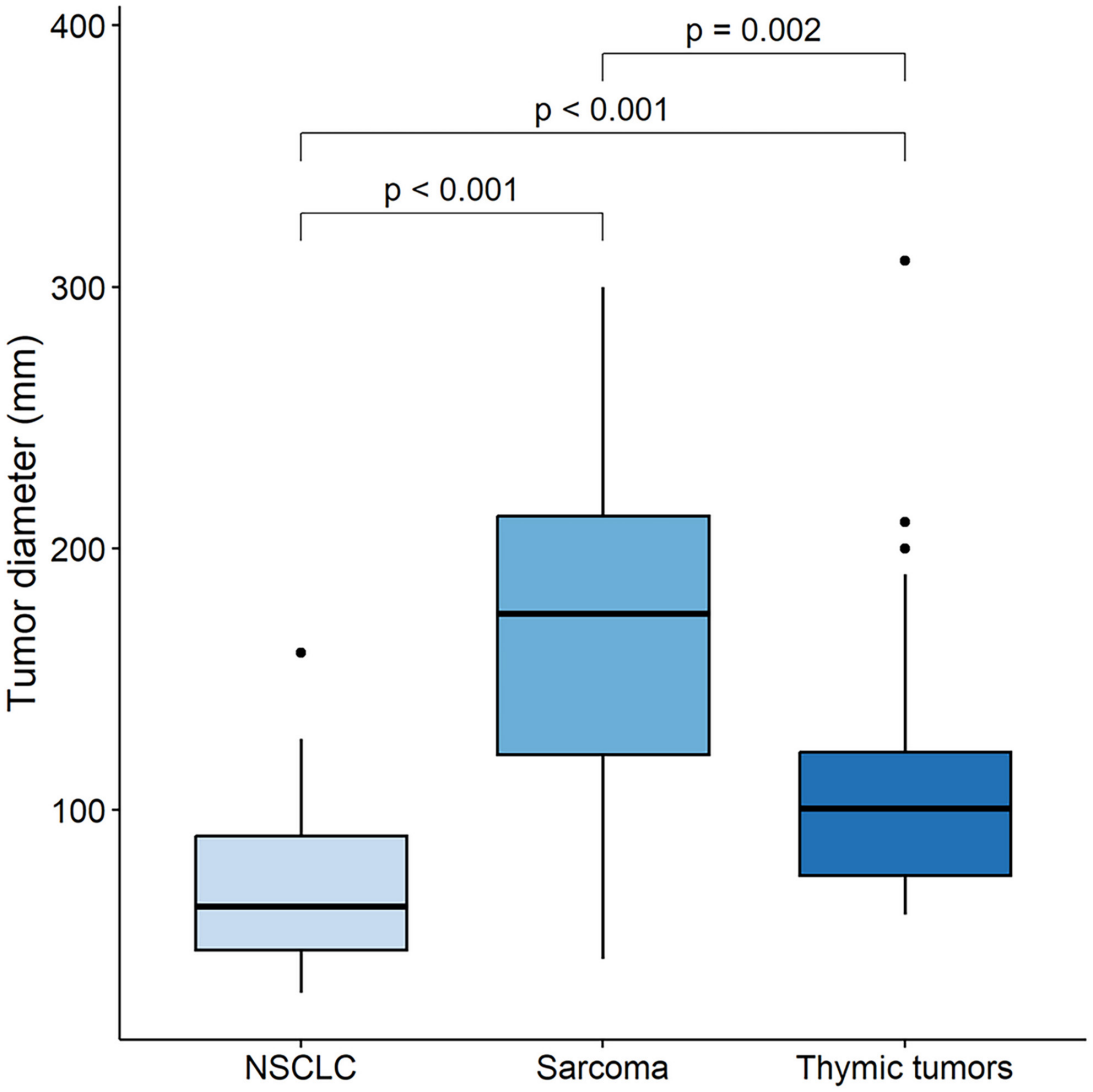
Boxplot showing the distribution of tumor diameters across the three most represented histologies. *p* values = Wilcoxon rank sum tests, corrected for multiple testing. NSCLC: Non‐small cell lung cancer.

**FIGURE 2:**
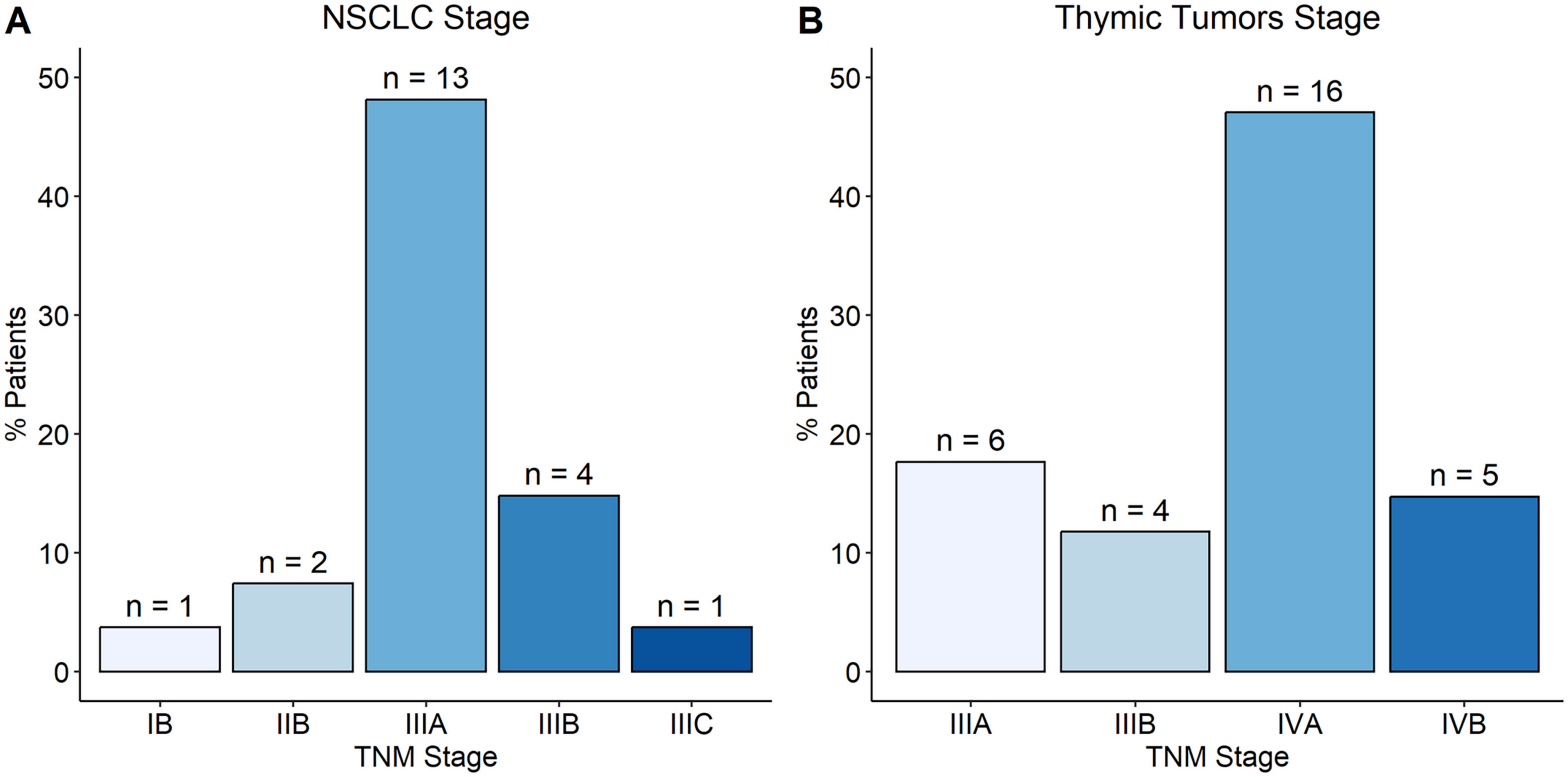
Disease stage distribution for thymic tumors and for non‐small cell lung cancer (NSCLC), according to their specific 8th TNM stage classification system.

During the study period, 91 patients were operated on through a clamshell (*n* = 36) or a hemiclamshell (*n* = 55) incision. Table 1 shows the baseline clinical characteristics of the study cohort. The majority of patients (57.1%) underwent neoadjuvant therapy, consisting in chemotherapy in 47 cases and chemo‐radiotherapy in 5. Thymic neoplasms (including thymomas in 31 cases and thymic carcinomas in 3 cases), non‐small cell lung cancer (NSCLC), and primary intrathoracic sarcomas were the most represented tumor types (37.4%, 29.7%, and 17.6%, respectively). Sarcomas were more common in the clamshell group and thymic neoplasms in the hemiclamshell group. Tumors in the clamshell group were prevalently located in one hemithorax (41.7%) or in the middle/posterior mediastinum (38.9%), while those in the hemiclamshell group were mainly located in one hemithorax (65.5%) or in the anterior mediastinum (30.9%, *p* < 0.001). The median tumor diameter was 100 mm (IQR: 64–140 mm), and was not significantly different between patients approached by clamshell or hemiclamshell incisions (Table 1). However, median tumor size was significantly different between sarcomas, thymic tumors and NSCLC (median size: 175 [IQR: 121–212] mm for sarcomas, 100 [IQR: 75–122] mm for thymic tumors, and 63 [IQR: 46.5–90] mm for NSCLC, Figure 1). Figure 2 shows the pathologic stage distribution for lung cancer and thymic neoplasms. The most common disease stage was IVA for thymic neoplasms (47.1%) and IIIA for NSCLC (48.1%).

**TABLE 1:** Baseline patients' characteristics.

Characteristic	Overall (*n* = 91)	Clamshell (*n* = 36)	Hemiclamshell (*n* = 55)	*p*
Gender				0.416
Female	35 (38.5%)	12 (33.3%)	23 (41.8%)	
Male	56 (61.5%)	24 (66.7%)	32 (58.2%)	
Age (years)	59 (49–65)	54 (42–65)	60 (50–65)	0.424
ASA score				0.827
2	39 (42.9%)	17 (47.2%)	22 (40.0%)	
3	47 (51.6%)	17 (47.2%)	30 (54.5%)	
4	5 (5.5%)	2 (5.6%)	3 (5.5%)	
Previous thoracic surgery	28 (32.9%)	7 (23.3%)	21 (38.2%)	0.164
Neoadjuvant therapy	52 (57.1%)	17 (47.2%)	35 (63.6%)	0.122
Tumor diameter (mm)	100 (64–140)	115 (60–200)	90 (65–112)	0.182
Histology				**< 0.001**
Thymic	34 (37.4%)	7 (19.4%)	27 (49.1%)	
Lung	27 (29.7%)	9 (25.0%)	18 (32.7%)	
Sarcoma	16 (17.6%)	12 (33.3%)	4 (7.3%)	
Germ cell	6 (6.6%)	2 (5.6%)	4 (7.3%)	
Trachea	2 (2.2%)	2 (5.6%)	0 (0.0%)	
Other	6 (6.6%)	4 (11.1%)	2 (3.6%)	
Prevalent lesion extension				**< 0.001**
Hemithorax	51 (56.0%)	15 (41.7%)	36 (65.5%)	
Anterior mediastinum	24 (26.4%)	7 (19.4%)	17 (30.9%)	
Middle/post mediastinum	16 (17.6%)	14 (38.9%)	2 (3.6%)	

*Note:* Numerical data are reported as median (IQR). Valuables in bold indicates statisitcal significance with *p* < 0.05.

Abbreviation: ASA, American society of anesthesiologists.

**FIGURE 3:**
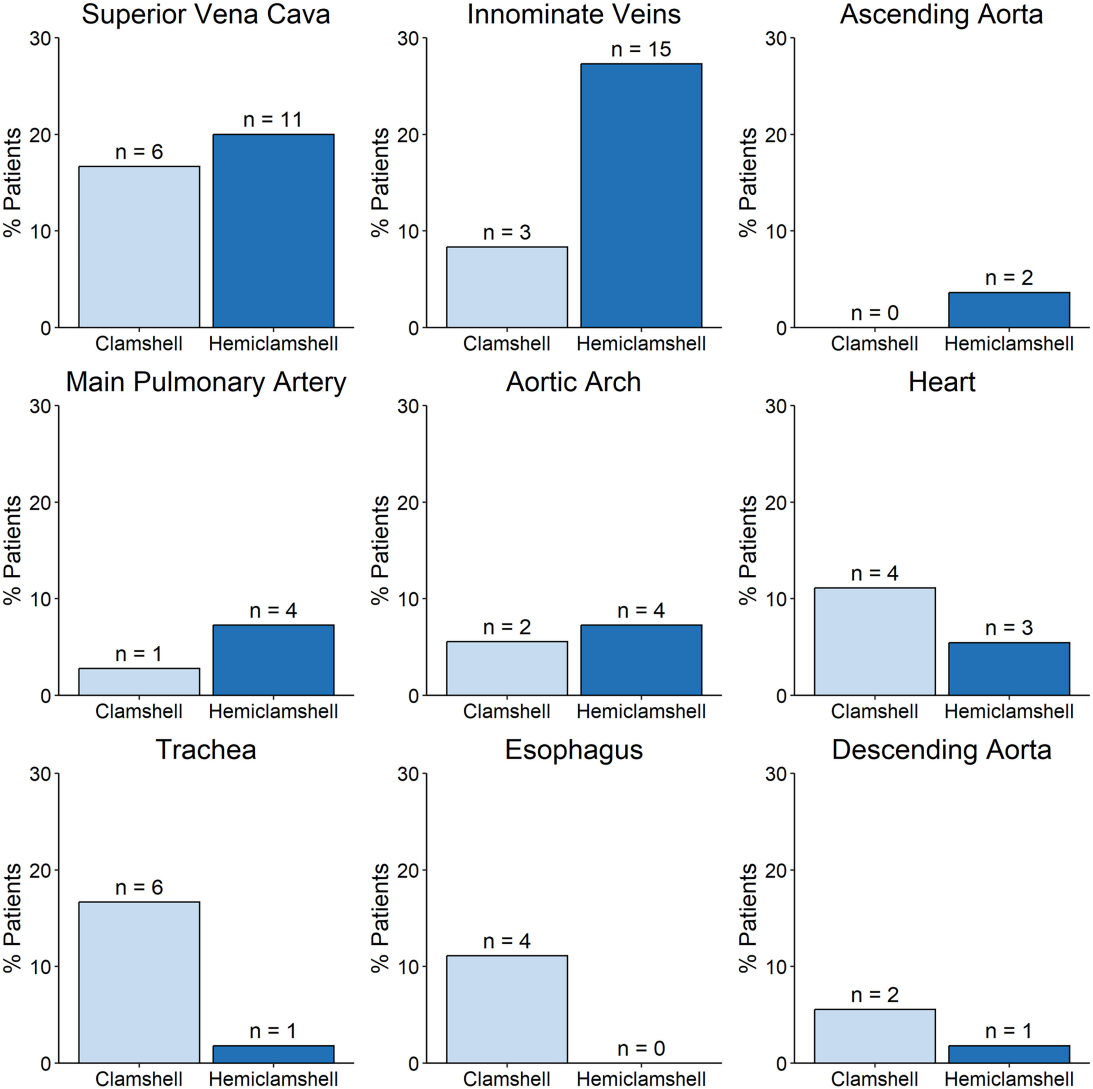
Overview of the proportion of patients who required resection of the different mediastinal structures. Panels are arranged row‐wise from top left to bottom right, following the anatomical position of the structures from anterior to posterior. Subadventitial aortic resections accounted for 7 of 11 aortic resections, while resections of the left atrium were performed in 3 of 6 cases using proximal clamping without cardiopulmonary bypass.

Regarding intraoperative characteristics (Table 2), 29/91 patients underwent pneumonectomy (41.4%). The pneumonectomy rate was higher in the clamshell than in the hemiclamshell group (64.0% vs. 28.9%, respectively). The vascular resection rate was 33.0%, and it was similar between the two groups, with vascular conduit interposition as the most common type of reconstruction (70.0%). Tracheo‐carinal resections were more common in the clamshell group (6/36, 16.7%), while pleural resections were more common in the hemiclamshell group (17/55, 30.9%). Overall, 10 patients (11%) underwent some form of extracorporeal support, including cardiopulmonary bypass (CPB, *n* = 8) and extracoroporeal membrane oxygenation (ECMO, *n* = 2). Extracorporeal support was used in eight cases for resection of heart/great vessels and two cases for maintenance of oxygenation during tracheo‐carinal resections. Figure 3 provides an overview of the proportion of patients who required resection of the different mediastinal structures (sorted by their anatomic position, from anterior to posterior).

**TABLE 2:** Intraoperative and postoperative variables.

Characteristic	Overall (*n* = 91)	Clamshell (*n* = 36)	Hemiclamshell (*n* = 55)	*p*
Lung resection (any)	70 (76.9%)	25 (69.4%)	45 (81.8%)	0.171
Lung resection type				**0.018**
Wedge/segment	13 (18.6%)	3 (12.0%)	10 (22.2%)	
Lobectomy/bilobectomy	28 (40.0%)	6 (24.0%)	22 (48.9%)	
Pneumonectomy	29 (41.4%)	16 (64.0%)	13 (28.9%)	
Vascular resection	30 (33.0%)	11 (30.6%)	19 (34.5%)	0.692
Vascular reconstruction type				0.851
Conduit	21 (70.0%)	7 (63.6%)	14 (73.7%)	
Patch	4 (13.3%)	2 (18.2%)	2 (10.5%)	
Suture	5 (16.7%)	2 (18.2%)	3 (15.8%)	
Tracheobronchial resection				**0.030**
No	80 (87.9%)	29 (80.6%)	51 (92.7%)	
Bronchial	4 (4.4%)	1 (2.8%)	3 (5.5%)	
Tracheo‐carinal	7 (7.7%)	6 (16.7%)	1 (1.8%)	
Pleural resection	19 (20.9%)	2 (5.6%)	17 (30.9%)	**0.004**
Resection status^a^				0.678
R0	72 (79%)	31 (86.1%)	41 (89.1%)	
R1	10 (11%)	5 (13.9%)	5 (10.9%)	
Extracorporeal support	10 (11.0%)	5 (13.9%)	5 (9.1%)	0.509
Extracorporeal support type				0.444
CBP	8 (80.0%)	3 (60.0%)	5 (100.0%)	
ECMO	2 (20.0%)	2 (40.0%)	0 (0.0%)	
Operative time (min)	300 (240–360)	310 (242.5–360)	300 (228–360)	0.475
Blood loss	675 (300–1.400)	750 (400–1500)	600 (300–1300)	0.529
Blood transfusions	52 (57.1%)	22 (61.1%)	30 (54.5%)	0.536
Intraop inotropes	41 (45.1%)	19 (52.8%)	22 (40.0%)	0.231
Postoperative ICU	86 (94.5%)	35 (97.2%)	51 (92.7%)	0.645
ICU stay (days)	3 (1–4)	3 (2–4.5)	3 (1–4)	0.231
Postop tracheostomy	10 (11.0%)	5 (13.9%)	5 (9.1%)	0.509
Postop inotropes	32 (36.0%)	15 (44.1%)	17 (30.9%)	0.207
Reoperation	11 (12.2%)	5 (13.9%)	6 (11.1%)	0.749
Major complications	21 (23.1%)	9 (25.0%)	12 (21.8%)	0.725
Postopstay (days)	12 (8–18)	13 (10.5–18.5)	11 (8–16)	**0.045**
In‐hospital mortality	4 (4.4%)	2 (5.6%)	2 (3.6%)	0.647
30‐day readmission	5 (5.7%)	1 (3.0%)	4 (7.3%)	0.646
90‐day mortality	6 (6.6%)	4 (11.1%)	2 (3.6%)	0.209

*Note:* Numerical data are reported as median (IQR). Valuables in bold indicates statisitcal significance with *p* < 0.05.

Abbreviations: CBP, cardiopulmonary bypass; ECMO, extracorporeal membrane oxygenation; ICU, intensive care unit.

^a^ Excludes 9 patients with pleural dissemination (resection status not assessable).

The median operative time was 300 min (IQR: 240–360 min), and it was similar between the two groups. A macroscopically complete resection was achieved in all patients; however, microscopic residual disease (R1) was identified in 10 patients (11%), while in 9 patients with pleural dissemination the resection status could not be assessed. Median duration of postoperative hospital stay was longer in the clamshell than in the hemiclamshell group (13 vs. 11 days, respectively), while all other early outcomes including blood loss, blood transfusions, intraoperative or postoperative inotropes, intensive care unit (ICU) stay, major morbidity, 30 and 90 days mortality rates were comparable (Table 2). No patient in the clamshell or hemiclamshell group experienced complications directly related to the surgical incision (e.g., wound infection, dehiscence or malunion), complications are reported in Table S1 (Online Resource 1).

**FIGURE 4:**
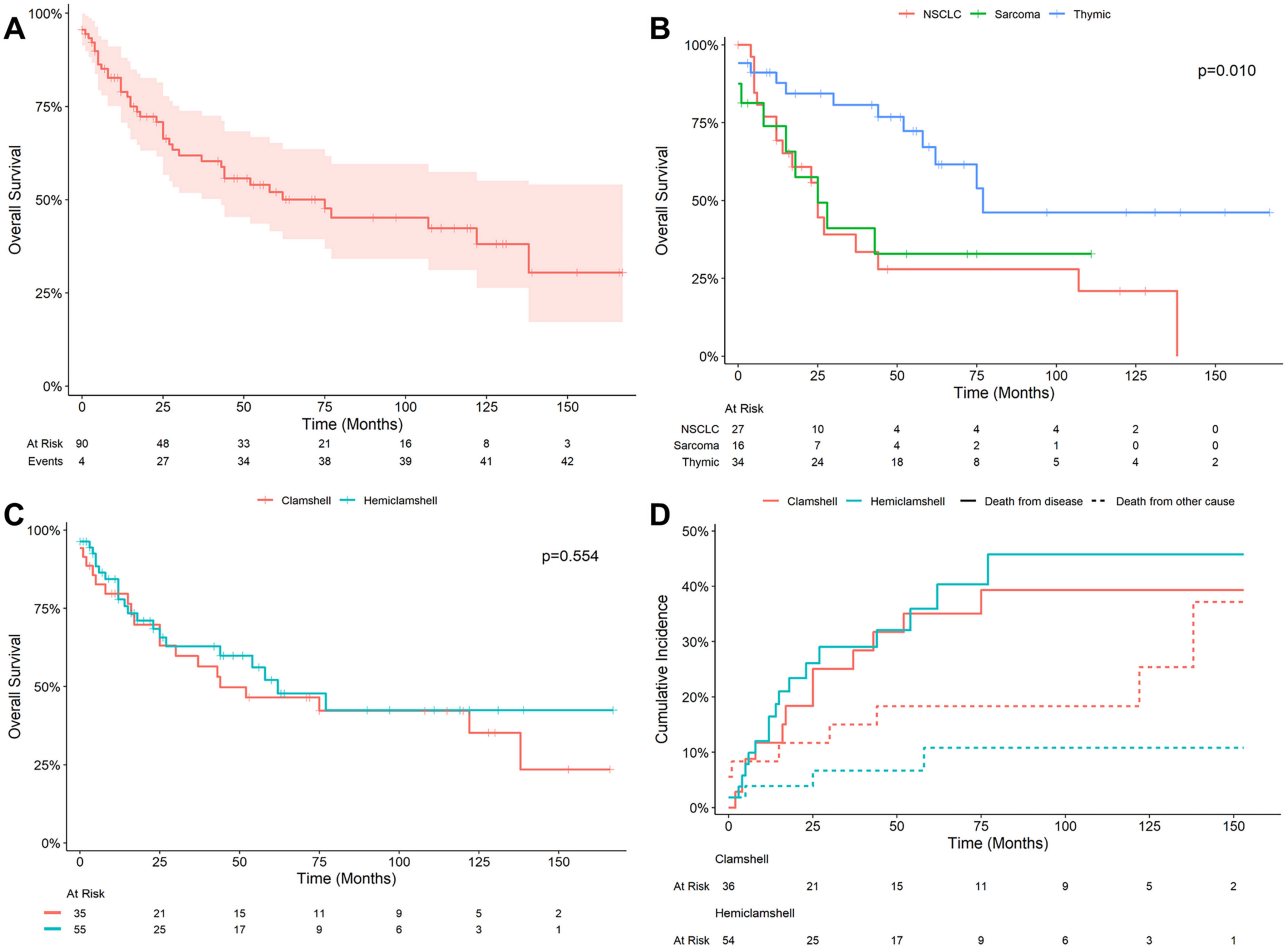
Overall survival. (A) Total population. (B) Patients affected by thymic tumors, lung cancer or sarcoma. (C) Overall survival according to the type of incision (D) Cumulative incidence of cause‐specific death (death from disease and death from other cause) according to the type of incision.

After a median follow‐up time of 71 (95% CI: 55–115) months, 42 patients died, including 29 from cancer‐related causes, 12 from other causes, and one of unknown cause. Other causes of death included cardiovascular events (*n* = 2), respiratory failure (*n* = 4), infections (*n* = 4), and stroke (*n* = 1). The 1‐, 2‐, and 5‐year OS rates (95% CI) in the whole cohort were 78.9% (70.8%–88.1%), 71% (61.5%–81.5%), and 52% (41.5%–65.2%), respectively (Figure 4A). OS was significantly better for patients affected by thymic tumors compared with NSCLC or sarcoma (Figure 4B, log‐rank test: *p* = 0.01). At competing risks analysis, the cumulative incidence of death from disease and death from other causes were 14.5% (95% CI: 7.9%–23.1%) and 5.7% (2.1%–11.9%), respectively at 12 months; 22.9% (14.2%–32.8%) and 7.0% (2.8%–13.8%), respectively, at 24 months; and 35.9% (24.7%–47.2%) and 13.6% (6.8%–22.9%), respectively, at 5 months. The OS rates were not significantly different between the two incisions (log‐rank test *p* = 0.554, Figure 4C). Similarly, the cumulative incidence of death from disease and death from other causes between the two incisions were comparable (Gray's test: death from disease *p* = 0.681, death from other cause *p* = 0.128, Figure 4D).

## 5 Discussion

**FIGURE 5:**
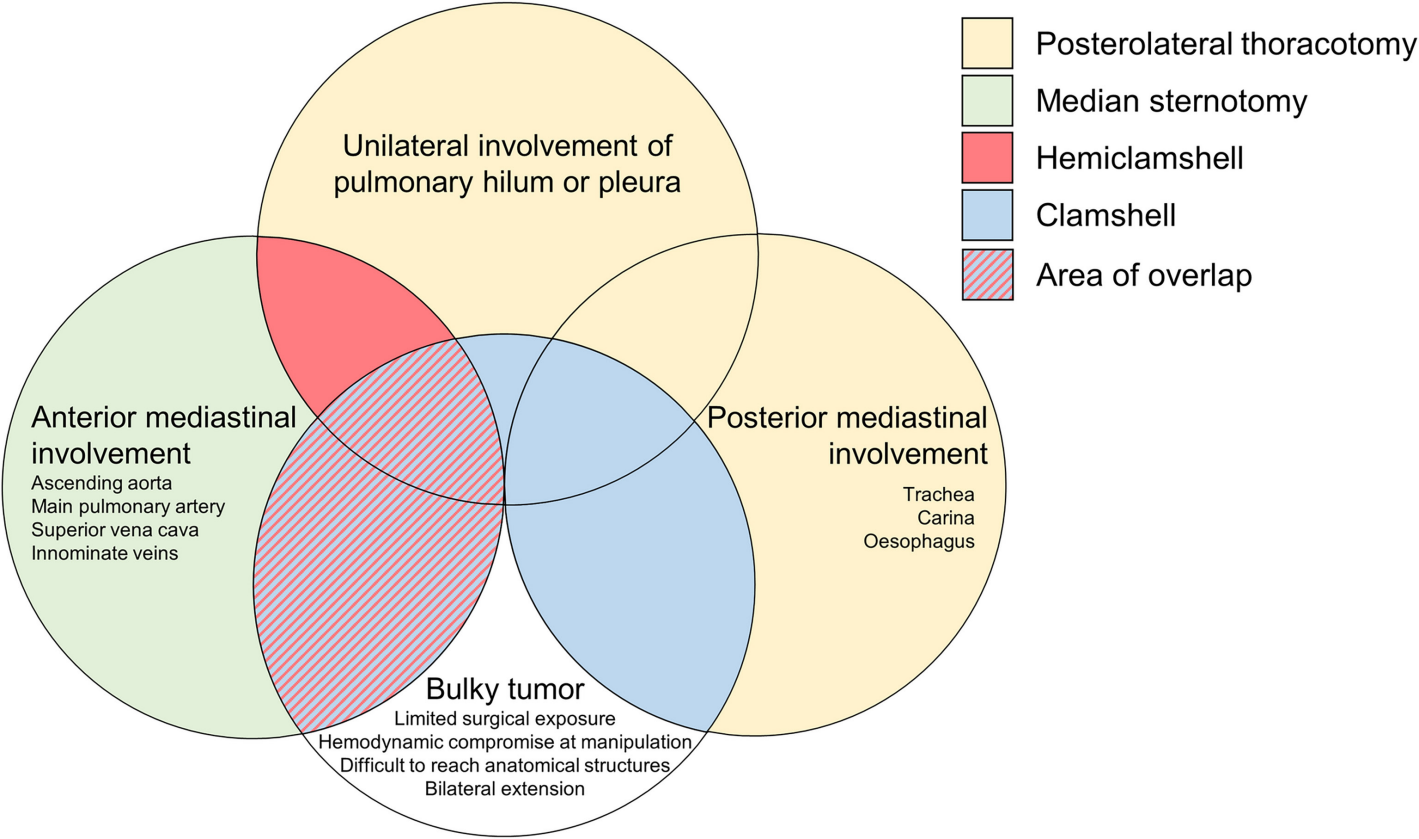
Venn diagram showing criteria for selecting different thoracic incisions. Circles represent specific intrathoracic tumor conditions, including involvement of anatomical regions/structures or presence of a bulky tumor. Intersections indicate overlapping conditions, where a more invasive approach (clamshell or hemiclamshell) may be required.

Clamshell and hemiclamshell approaches provide a wider surgical exposure of the chest than a standard median sternotomy or a posterolateral thoracotomy. They are generally reserved for particularly challenging cases, such as large tumors, close proximity of the tumor to vital, deeply located structures, or tumors that extend across different anatomical regions. A tumor may present with one or more of such characteristics at the same time, prompting the choice of a more extensive surgical approach. Therefore, criteria for selecting a more invasive thoracic incision may be illustrated by a Venn diagram (Figure 5), where each circle represents a specific tumor condition, such as the involvement of an anatomical region/structure or the presence of a bulky tumor. It is when these conditions overlap that a thoracotomy or a median sternotomy may not be sufficient. Certain areas of overlap are clearly specific for either the hemiclamshell or clamshell incisions; whereas in other cases the choice between the two approaches should be made on a case‐by‐case basis.

Consistent with the conceptual framework illustrated in Figure 5, retrospective analysis of the present series shows that patients undergoing clamshell or hemiclamshell incisions share several common features, including a large median tumor size, a relatively high rate of vascular resections, and long median operative time. At the same time, distinct clinical patterns specific to each surgical approach were identified.

The clamshell incision allows an excellent, bilateral exposure of the structures of the posterior mediastinum, including the distal trachea/carina and the esophagus. In standard cases, both these anatomical structures can be reached by a right thoracotomy; however, if the tumor is large, or it extends toward the contralateral left side, if there's a need to control the great vessels or to displace the heart, in all these cases a clamshell approach may be preferable. Our descriptive analysis reflects in part these considerations, as tumors located in the posterior mediastinum were predominantly approached with a clamshell incision (38.9% and 3.6%, in the clamshell and hemiclamshell groups, respectively). Moreover, this approach was used more frequently to perform tracheo‐carinal resections (16.7% vs. 1.8%, respectively). Notably, the majority of the tracheo‐carinal resections in the clamshell group (four of six) were associated with left‐sided lung resections (pneumonectomy in three cases and upper lobectomy in one). In fact, left‐sided tracheobronchial tumors that involve the carinal bifurcation represent a particular technical challenge due to the anatomical relationship between the aortic arch and the left tracheobronchial angle, and the clamshell incision is traditionally mentioned among the viable options to approach these cases [15, 16, 17].

The hemiclamshell incision is well suited for tumors extending between the anterior mediastinum and one hemithorax. This anatomical pattern typically results either from thymic tumors spreading laterally toward the pulmonary hilum or pleura, or from lung cancers extending medially toward the great vessels (e.g., the ascending aorta or the main pulmonary artery). In fact, thymic tumors and lung cancers were largely the two most represented tumor types in the hemiclamshell group (49.1% and 32.7%, respectively). Moreover, many of the thymic tumors were stage IVa tumors with pleural metastases, which explains the high rate of pleural resections observed in the hemiclamshell group (30.9%). Other authors have found the hemiclamshell approach convenient for thymic malignancies with pulmonary hilar invasion or pleural spread [9, 18], and for lung cancer invading the heart and great vessels [8].

There are only few case series in the literature describing the use of clamshell or hemiclamshell incisions for intrathoracic tumor resection, and all of them vary considerably in the characteristics of included patients. To the best of our knowledge, Bains et al. in 1994 published the largest case series of adult patients operated on through a clamshell (*n* = 71) or hemiclamshell (*n* = 19) incision [7]. In their study, the majority of patients underwent bilateral pulmonary wedge resections for metastatic disease (*n* = 62), an indication that is probably outdated since the introduction of minimally‐invasive thoracic surgery. The other patients were affected by large mediastinal tumors with extension into the thoracic cavity (*n* = 14), and primary pulmonary or pleural tumors with mediastinal involvement (*n* = 14), which is more comparable to the cases included in our study [7].

Several authors have reported on the use of the hemiclamshell incision to approach tumors of the cervicothoracic junction or superior sulcus tumors [12, 19, 20]. In some of these reports the hemiclamshell incision is extended to the neck, either along the anterior border of the sternocleidomastoid muscle or above the clavicle, so as to provide a wider exposure of the cervical region (the so called “trap‐door incision” described by Masaoka [21]). These studies are hardly comparable to the present one, since at our Center, we do not approach superior sulcus tumors or tumors of the cervicothoracic junction by hemiclamshell incision. For this indication we favor the anterior approach described by Grunenwald [22] or the posterior approach described by Paulson [23], depending on the case.

Overall, in the absence of comparable studies, the mortality and morbidity rates that we observed (4.4% and 23.1%, respectively) appear acceptable and proportionate to the complexity of the procedures. Because it is evident that patients in the clamshell and hemiclamshell groups have different characteristics and different primary diseases, it is not the surgical approach per se the main determinant of outcomes. Therefore, only descriptive comparisons can be presented.

The majority of early outcome measures, including the duration of ICU stay, need for postoperative inotropes and major morbidity rate were not significantly different between the two incisions; however, patients in the clamshell group had a longer postoperative stay. This finding may be explained by the higher pneumonectomy rate in the clamshell than in the hemiclamshell group (64% vs. 28.9%, respectively).

Long‐term survival outcomes in this heterogeneous group of patients should be interpreted in light of the ominous tumor characteristics, including their large median size and the proportion of advanced stage tumors (Figure 1). Unsurprisingly, thymic malignancies were associated with a longer OS (67% at 5 years, 95% CI: 51%–88%) compared to lung cancer or sarcoma (Figure 4b). This is in agreement with the relatively indolent course of disease of thymic tumors. The OS rates herein observed for the thymic tumors compare favorably with other large cohort studies of advanced stage thymic tumors, unselected for their surgical approach [24, 25]. Concerning NSCLC or sarcomas, the unique patients' subset and the heterogeneity in the stage of disease makes it inappropriate to make comparisons with any other reference cohort.

Over the long term, mortality due to cancer occurred more than twice as often as death for other reasons, which may potentially include sequelae from the surgical procedure. Consistent with this interpretation, the curve of the cumulative incidence of non‐cancer‐related death seemed to reach a plateau at 60 months after surgery (Figure 4d), suggesting that long‐term survival beyond that point is mainly determined by the underlying tumor prognosis.

## 6 Conclusions

This study represents one of the largest case series evaluating the use of clamshell and hemiclamshell incisions for the management of complex intrathoracic tumors. In this series, both approaches were employed to treat highly challenging conditions, including large tumors and cases requiring pneumonectomy, vascular or tracheobronchial resections, or extracorporeal support.

The clamshell incision was predominantly adopted for tumors involving the posterior mediastinum and for tracheo‐carinal resections, whereas the hemiclamshell approach was mainly used for lesions extending between the anterior mediastinum and one hemithorax, such as advanced thymic malignancies with pulmonary hilar invasion or pleural dissemination, and lung cancer with proximal involvement of the great vessels.

Although clear‐cut indications for either incision cannot be established, we propose a conceptual framework to assist in identifying clinical scenarios that may warrant the use of these more invasive surgical approaches (Figure 5). Surgical outcomes should be interpreted in light of the complexity of the procedures and the unfavorable tumor characteristics; nevertheless, both early and late results appear acceptable in this challenging patient population. Direct comparisons with other retrospective series remain limited by the paucity of similar reports and by heterogeneity in surgical indications.

These findings may contribute to a more rational selection of surgical access in patients with complex intrathoracic tumors requiring extended resection.

## Author Contributions

**Alessandro Bonis:** data curation, investigation, methodology. **Federico Rea:** writing – review and editing, conceptualization, project administration, supervision, visualization, validation. **Viola Sambataro:** data curation, investigation, methodology. **Vincenzo Verzeletti:** conceptualization, methodology, investigation, writing – original draft, data curation. **Andrea Dell'Amore:** writing – review and editing, visualization, conceptualization, supervision, project administration, validation. **Marco Mammana:** conceptualization, formal analysis, data curation, writing – original draft, methodology, investigation, software. **Giulia Pagliarini:** data curation, investigation, methodology.

## Funding

The authors have nothing to report.

## Conflicts of Interest

The authors declare no conflicts of interest.

## Supporting information


**Table S1:** Overview of postoperative complications in the clamshell and hemiclamshell groups.

## Data Availability

The data that support the findings of this study are available from the corresponding author upon reasonable request.
