# Supplementary material for: Clamshell and Hemiclamshell Incisions for Complex Intrathoracic Tumors: A Retrospective Analysis of Indications and Outcomes
Source: Thorac Cancer. 2026 Aug 18;17(16):e70373. doi: 10.1111/1759-7714.70373 (PMC13483318; doi:10.1111/1759-7714.70373)
Supplement: Supplementary file 1 [file TCA-17-e70373-s001.docx]

**Table S1:** Overview of postoperative complications in the clamshell and hemiclamshell groups

| **Post-operative complications** | **Overall**  **(n = 91)** | **Clamshell**  **(n = 36)** | **Hemiclamshell**  **(n = 55)** |
| --- | --- | --- | --- |
| Atrial fibrillation | 15 (16.5 %) | 4 (11.1 %) | 11 (20 %) |
| Left recurrent nerve paralysis | 13 (14.3 %) | 6 (16.7 %) | 7 (12.7 %) |
| Haemothorax | 10 (10.9 %) | 4 (11.1 %) | 6 (10.9 %) |
| Deep vein thrombosis and/or pulmonary embolism | 9 (9.8 %) | 3 (8.33 %) | 6 (10.9 %) |
| Pneumonia | 4 (4.4 %) | 4 (11.1 %) | 0 (0 %) |
| Stroke | 3 (3.3 %) | 3 (8.33 %) | 0 (0 %) |
| Respiratory failure | 3 (3.3 %) | 1 (2.77 %) | 2 (3.6 %) |
| Right recurrent nerve paralysis | 1 (1.1 %) | 1 (2.77 %) | 0 (0 %) |
| Multiple organ failure | 1 (1.1 %) | 1 (2.77 %) | 0 (0 %) |
| Cardiac arrest | 1 (1.1 %) | 0 (0 %) | 1 (1.8 %) |
| Diaphragmatic hernia | 1 (1.1 %) | 0 (0 %) | 1 (1.8 %) |
| Thrombosis of the vascular prosthesis | 1 (1.1 %) | 0 (0 %) | 1 (1.8 %) |
| Hydropneumothorax | 1 (1.1 %) | 0 (0 %) | 1 (1.8 %) |
| Left phrenic nerve paralysis | 1 (1.1 %) | 1 (2.77 %) | 0 (0 %) |
